# Color Stability Enhancement and Antioxidation Improvement of Sanhua Plum Wine under Circulating Ultrasound

**DOI:** 10.3390/foods11162435

**Published:** 2022-08-13

**Authors:** Zhiqian Wu, Xusheng Li, Yingyu Zeng, Dongbao Cai, Zhaojun Teng, Qixia Wu, Jianxia Sun, Weibin Bai

**Affiliations:** 1 Department of Food Science and Engineering, Institute of Food Safety and Nutrition, Guangdong Engineering Technology Center of Food Safety Molecular Rapid Detection, Jinan University, Guangzhou 510632, China; 2 School of Chemical Engineering and Light Industry, Guangdong University of Technology, Guangzhou 510006, China

**Keywords:** ultrasonic, Sanhua plum, wine, anthocyanins, color, antioxidant

## Abstract

Anthocyanins contribute to the attractive color of fruit wine, and their excessive degradation is deleterious to quality, especially for wine with an inherently low anthocyanin content, such as Sanhua plum wine. Ultrasonic treatment is well recognized for wine color maintenance. In the present study, fresh Sanhua plum wine was ultrasonic-treated and aged in barrels for three months. Our results demonstrate that ultrasonic treatment at 28 and 40 kHz improves color performance, as expressed by an increase in a*, b*, and C* values and color intensity, which is highly related to copigmentation. This successful conservation was attributed to the inactivation of polyphenol oxidase and the corresponding reduction in anthocyanin degradation. Finally, the increased antioxidative ability was verified due to the hydrogen donating ability of the surviving anthocyanins. This study indicates the reliability of ultrasonic treatment for providing superior colorfastness during Sanhua plum wine aging, which is also of great potential in processing different fruit wines.

## 1. Introduction

Sanhua plum (*Prunus salicina* Lindl) belongs to the *Rosaceae* family; it is the traditional fruit in southern China, and is ubiquitous throughout the world [1]. Plum ripening is characteristically accompanied by a gradually varied red coloration, both in skin and flesh, after flowering; this is attributed to anthocyanin synthesis, which is responsible for fruit quality and consumer preference [2]. However, the moisture content of the Sanhua plum reaches 80–90%, which provides a suitable microenvironment for microbial spoilage and chemical deterioration, and the high organic acid concentration renders the flesh an acerb flavor with regards to fresh fruit consumption [3]. Building on these facts, nearly 10–20% of plums worldwide are wasted per year [4]. Preferentially, given the features of high juice yield and acidity, fruit winemaking has recently been considered to resolve the issues of vulnerability and seasonality of Sanhua plums [5]. Additionally, Sanhua plum wine also comprises amino acids, mineral elements, and a myriad of polyphenols, alkaloids, and polysaccharides, contributing to excellent bioactivities [5].

Wine color performance is one of the crucial sensory quality properties and determinants of consumers purchase intention. Anthocyanins perform the dominating contributors to fruit wine color [6,7]. The chief drawback of using plums for winemaking is the relatively destitute anthocyanin contents, as well as the more unstable anthocyanin species compared to grapes, blueberries, and other anthocyanin-rich fruits [8]. These anthocyanins suffer degradation and absorption induced by the biological actions of yeast during alcoholic fermentation, followed by further anthocyanin derivative transformation in the presence of specific metabolites and phenolic acids during aging and storage [6]. Discrepancies in the observed effects of the color attenuation following plum wine fermentation and aging are significant, and directly threaten wine organoleptic quality, substantially shortening the shelf life [8]. Thus, it is vital to protect the intrinsic and restricted anthocyanins for a better color appearance. It has been well documented that the preferential selection and employment of non-*Saccharomyces* yeast can effectively reduce the expenditure of anthocyanins at the fermentation stage [9]. Meanwhile, physically accelerating aging under ultrasonic treatment was recently proposed as being beneficial to anthocyanin maintenance in real wine [10].

Ultrasonic treatment is frequently applied for wine aging to achieve a soft and smooth taste, which is attributed to its cavitation effects induced by changing aroma constituent profiles [11]. Mounting evidence illustrates that the effect of ultrasonic treatment on anthocyanins is heterogeneous in terms of the solution environment that the anthocyanins are exposed to. Unequivocally, ultrasonic-induced high energy, high temperature, and free radicals can destroy anthocyanins structures, especially in aqueous ethanol solutions [12,13,14]. Nevertheless, in the presence of precursors for anthocyanin derivative formation, ultrasonic treatment has the propensity to accelerate pyranoanthocyanin generation associated with the implementation of color stabilization [7,15]. This finding has updated our knowledge regarding the application of ultrasonic on color enhancement. More importantly, our previous study demonstrated a profound impact of ultrasonic treatment on the blueberry wine color stability and the corresponding desirability of an anthocyanin content safeguard [10]. However, the effects of ultrasonic treatment on anthocyanins in wine are uncertain in terms of structure polarity and treatment condition, resulting in a concern about the effectiveness and universality of ultrasonic treatment on diverse fruit wine systems in the aspect of anthocyanin protection. Since Sanhua plum wine exhibits entirely different anthocyanin profiles from other wines, associated with a low anthocyanin abundance, their tolerance to ultrasonic energy is problematic, and anthocyanin behaviors also remain ambiguous. It is of great interest to validate and explore the impact of ultrasonic treatment on Sanhua plum wine color features and anthocyanin changes during the aging period.

Overall, this study aimed to find complete evidence of the color stability of Sanhua plum wine, characterized by anthocyanin profiles after circulating ultrasonic treatment. This investigation also provides a novel method for the production of high-value Sanhua plum wine.

## 2. Material and Methods

### 2.1. Materials and Reagents

Sanhua plums were freshly gathered from an orchard in Heyuan, Guangdong, China. The *Saccharomyces cerevisiae* yeast used for alcoholic fermentation was purchased from Angel (Yichang, China). Acetonitrile and formic acid were the chromatography pure-grade substance used for high-performance liquid chromatography (HPLC), purchased from Merck (Darmstadt, Germany).

### 2.2. Fermentation of Sanhua Plum Wine

The fresh Sanhua plums were standardly cleaned, filtered, denucleated, and squeezed into juice using an equal mass of water. After enzymolysis and sugar adjustment, the unstrained juice, which had an initial Brix degree of 26, underwent alcoholic fermentation, as described in our previous study, at a laboratory scale [10]. *Saccharomyces cerevisiae* was first standardly activated for the alcoholic fermentation process with a ratio of 0.18% (*v*/*v*), and 50 mg/kg of potassium metabisulfite was employed for the purpose of antisepsis. The alcoholic fermentation was conducted in an incubator at a constant temperature of 25 °C for eight days, and post-fermentation was carried out at 10 °C, maintained for ten days. At the terminal process, 0.22 μm membrane filtration was employed to acquire the clarifying plum wine for the following ultrasonic treatments.

### 2.3. Ultrasonic Treatment

A plate-type ultrasonic producer (WKS400/4S, Five-pines UJS, Zhenjiang, China) was employed to ensure that the wine inside the instrument chamber equably receives sufficient ultrasonic treatment, which overcomes the limitations of the probe-type ultrasound. The new Sanhua plum wine samples were treated using a plate-type circulating ultrasonic agitating instrument at room temperature with rated power at 100 W for 20 min, and the ultrasonic frequency was independently set at 20, 28, and 40 kHz. Considering the chamber volume of the machine is 150 mL, every 3 L wine sample was circularly treated for 400 min to ensure each liquid sample was discontinuously processed for total 20 min. At a certain power, frequency, and time, a gap mode of 2 s running and 2 s stopping was set. After that, samples were stored in oak barrels for sampling once a week for the first month, and once a month for another two months. Each treatment was replicated three times.

### 2.4. Assessment of CIE-LAB Tristimulus Colors

The CIE-LAB tristimulus color of the wine was measured by spectrophotometer (CS-820N, CHN Spec., Hangzhou, China). The samples, in 12.4 × 12.4 × 45 mm quartz colorimetric cuvettes, were determined with a transmission code using distilled water as a control. Based on the color parameters of brightness (L*), redness (a*), and yellowness (b*), which were obtained under the circumstance of light source D65, and 10° angular measurement, the hue angle (*h_ab_*), chroma saturation (C*), and total color difference between control wine and wine treatments (∆*E*), were calculated using the following formulas and compared with the untreated sample at 0 weeks. All these measurements were repeated in triplicate.

*h_ab_* = arctan (b*/a*)

C* = [(a*)^2^ + (b*)^2^]^1/2^

∆*E* = [(∆L*)^2^ + (∆a*)^2^ + (∆b*)^2^]^1/2^

### 2.5. Determination of Color Intensity and Tonality

The absorbance of wine samples at wavelengths 420, 520, and 620 nm was measured by a spectrophotometer (GEN10S UV-Vis, Thermo Fisher Scientific, Waltham, MA, USA) using a 1 mm path length cell. The color intensity was described as the tenfold sum of absorbance at 620, 520, and 420 nm, and tonality (T) was defined as the ratio of absorbance at 420 nm and 520 nm.

### 2.6. Measurement of Anthocyanin Color Parameters

Anthocyanin color parameters are comprised of wine color due to pigments (WCP), wine color (WC), color due to pigment resistance to sulfur dioxide (CDR SO_2_), chemical age of wine (CAW), and free anthocyanins (FA) synthetically represent the color performance derived from anthocyanin profiles. Under an ultraviolet-visible spectrophotometer method, the measurement of the anthocyanins parameter of Sanhua plum wine was conducted referring to our previous research [10].

### 2.7. Individual Anthocyanin Identification and Quantification

A UPLC/MS-8045 liquid chromatography–electrospray mass spectrometer (Shimadzu Corporation, Kyoto, Japan) was employed to identify the anthocyanins in Sanhua plum wine. Referring to the indicated methods, with some modification, the collected Sanhua plum wine samples were previously filtrated through a 0.22 μm membrane, and a 2.0 μL sample was injected into a reversed-phase C18 column (2.1 × 100 mm, 1.8 μm, Phenomenex, Tianjin, China) for component separation maintained at a flow rate of 0.2 mL/min. For the program, 2% formic acid, by gradient, and acetonitrile were used as solvent A and B, respectively, and the gradient program followed the description in our previous study [10]. The full mass scan was in the range of 100–1500 m/z in a positive ion mode subjected to electrospray ionization, and the mass parameter was also set according to our previous study [10]. The subsequent quantification of anthocyanins was implemented using an HPLC system (Thermo Fisher Scientific), as our research described previously [10].

### 2.8. Polyphenol Oxidase and Peroxidase Activity Assessment

Polyphenol oxidase (PPO) and peroxidase (POD) activity of Sanhua plum wine was analyzed by commercial kits from Nanjing Jiancheng Bioengineering Institute following the standard protocol. PPO activity assessment was based on the ability of PPO to catalyze the substrate into quinones characterized by an absorption of 420 nm. Meanwhile, the POD activity was defined as the catalytic reaction targeted to H_2_O_2_, in which the absorption at 420 nm was the determined wavelength as well.

### 2.9. Antioxidative Properties Evaluation

The produced wine samples, after three-month storage, were subjected to antioxidative properties assessment concerning the scavenging ability of 1-diphenyl-2-picrylhydrazyl free radical (DPPH·), superoxide anion free radical (O^2−^·), and 2,2′-azinobis-(3-ethylbenzthiazoline-6-sulphonate) cationic free radical (ABTS^+^·), following the description in the published article [10].

### 2.10. Data Analysis

All trials were conducted in triplicate. The statistical analysis of changes in color performance, anthocyanin color value, anthocyanin level, and antioxidative ability were carried out using one-way ANOVA, with Tukey’s multiple comparisons test as a post hoc comparison, using SPSS 25.0 (IBM^®^ SPSS^®^ Statistics, Chicago, IL, USA). Data are presented as mean ± standard deviation, and statistical significance was considered at *p* < 0.05. The data were visualized using GraphPad Prism 8.0 (GraphPad, San Diego, CA, USA).

## 3. Results and Discussion

### 3.1. Ultrasonic Treatment Slows Wine Decoloration

Low-frequency ultrasonic treatment for food processing ranges from 20 kHz to 100 kHz [16]; more specifically, it has been reported that the frequency of 20 kHz to 40 kHz is an advisable range for winemaking or stabilized anthocyanin generation [17,18]. The probe- and plate-type ultrasonic treatments are two dominant modes, and are suitable for different scenarios. Compared to the probe-type ultrasonic treatment, the plate-type mode preserves the unique dispersibility and uniformity of energy, and is more appropriate for wine production. In the present study, a plate-type circulating ultrasonic producer was employed, and provided a specific ultrasonic frequency at 20 kHz, 28 kHz, and 40 kHz. Meanwhile, the cyclical mode also conferred to the liquids intensive mixing and local overheating prohibition.

**Figure 1:**
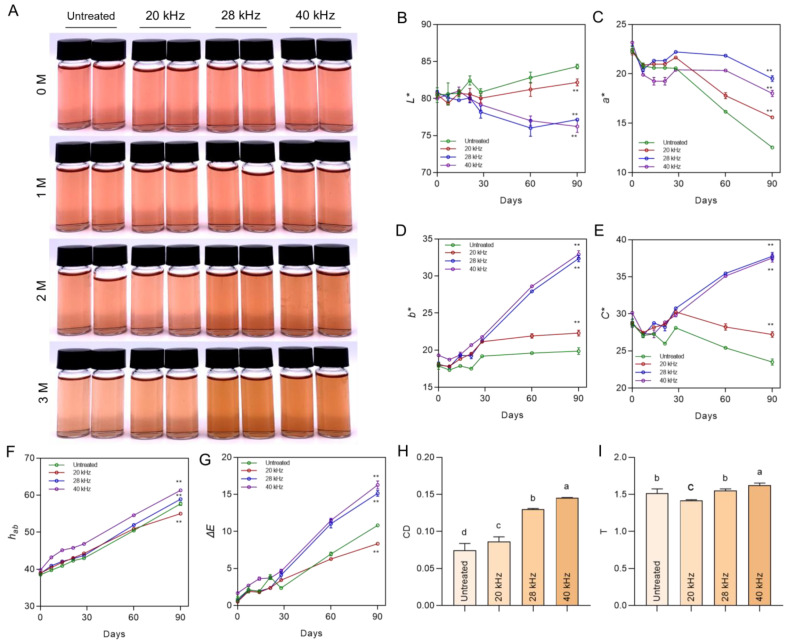
Color characteristic index of Sanhua plum wine during the whole aging period. (**A**) Representative image of fermentation solution. The images were captured with the same camera parameters under uniform lighting conditions. (**B**–**G**) CIE-LAB tristimulus color assessment results. (**H**) The color intensity of three-month aging samples. (**I**) Color tonality of three-month aging samples. ** *p* < 0.01, compared with untreated wine. For color intensity and color tonality, different letters indicate a significant difference between the groups.

Wine storage in oak barrels is a traditional practice in winemaking worldwide. Although there are many alternative containers, the oak barrel remains one of the most popular options in wine production when considering its positive influence on the organoleptic quality and the complexity of wine [19]. During wine aging, undesired wine decoloration is chiefly attributed to anthocyanin degradation and transformation moderated by complex mechanisms. Monitoring the wine color performance at the given time points within the aging period in an oak barrel, a degenerative red color was predominantly observed, and a yellowish hue gradually appeared during the three-month aging period without ultrasonic treatment, as shown in Figure 1A. This natural change was derived from micro-oxygenation, oak wood phenolic extraction, and the interactions between wine phenolics and oak wood extractives. Interestingly, ultrasonic treatment dramatically played a constructive role in achieving a strong redness reservation that was close to the original fresh wine. The frequency was obviously involved in effecting color persistence; the treatment frequency at 20 kHz was equipped with fine-tuning, while a higher frequency corresponds to a more attractive color. This visible color reinforcement was first discovered after two-month aging, and was intensified in the three-month sample.

**Figure 2:**
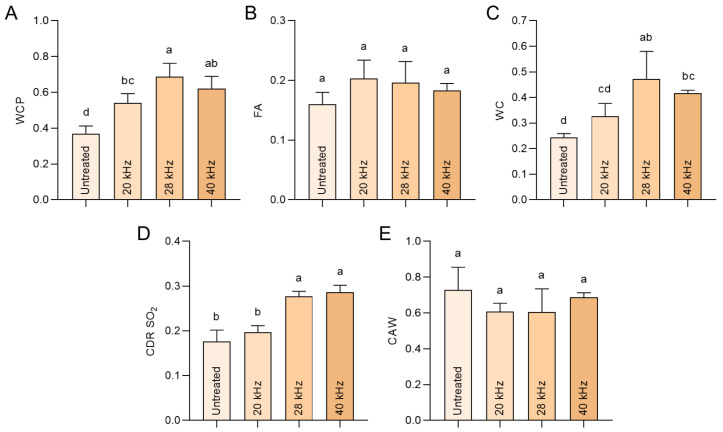
Anthocyanin color values of wine. The color values originating from anthocyanins were determined in the wine after three months of aging. (**A**) Wine color due to pigments (WCP). (**B**) Free anthocyanins (FA). (**C**) Wine color (WC). (**D**) Color due to pigment derivatives resistant to SO_2_ (CDR SO_2_). (**E**) Chemical age of wine (CAW). Different letters indicate a significant difference between the groups.

CIE-LAB tristimulus colors can comprehensively quantify the color characteristics, for which the values of L*, a*, b*, C*, and *hab* represent lightness (bright to dark), color from green to red, color from blue to yellow, chroma, and hue angle, respectively. These color parameters were obtained as shown in Figure 1B–G. The untreated wine expressed a continuous reduction in the values of a* and C* (Figure 2C,E), and a rising tendency in L*, b*, and *hab* values (Figure 2B,D,F) during the three-month storage period, directly giving rise to the noticeable increases in chromatic aberration (∆*E*) (Figure 2G), sharply contrasting with the newly fermented wine. Generally, the decoloration widely exists in wine aging in response to pigment degradation and oxidization [20]. The relevant refinement stage of wood barrel aging compared to bottle aging, oxygen entry, phenol migration from the barrel, complexation of pigments with matrixes, and copigmentation are all involved in the color aberrance [21]. These factors conjointly participate in the intensive yellowish color formation that is considered the symbol of wine maturity, and highly recommended in aged wine [22]. The ultrasonic treatments under different conditions all positively resulted in the desired color presentation associated with surpassing values for both a* and b*, representing reduced red color loss as well as yellow pigment generation, which is consistent with the sensory recognition presented in Figure 1A. Additionally, reversed saturation after 28 kHz and 40 kHz ultrasonic treatment was extensive, and indicated strong evidence of brilliant color with attractiveness in line with the b* value, which progressively faded in the untreated and 20 kHz treated groups (Figure 2D). An Δ*E* value of 3 represents the theoretical limit of perception for the human eye [10]. In this study, ultrasonic treatment under 28 kHz and 40 kHz both resulted in values of Δ*E* greater than 15 after three months compared to the baseline, whereas this value for the untreated group was less than 8 (Figure 1G). This difference between the ultrasonic treated groups and untreated group demonstrated that there is an observable color change in the human eyes, which is consistent with Figure 1A. Strictly speaking, throughout the period studied, a higher frequency predisposes the wine body to better color stability, and this enhancement approached beneficial levels when the frequency was close to 40 kHz. This concept is also evidenced by the visible color presentation comparing 28 kHz and 40 kHz (Figure 1A).

Collectively, after ultrasonic treatment, the Sanhua plum wine samples possessed stronger color stability and reduced decoloration during oak barrel aging. Similar protective effects that profit from ultrasonic have also been documented in blueberry wine [10] and mulberry wine [23], after proper treatment.

### 3.2. Ultrasonic Treatment Influences Color Intensity and Color Tonality

Color intensity partly reflects the reduction in monomeric anthocyanin concentrations and the formation of anthocyanin derivatives in fruit wine [24]. As expected, the predominantly amplified CD value was observed in the ultrasonic-assisted Sanhua plum wine samples (Figure 1H). This beneficial effect also existed in ultrasonic-processed mulberry wine, suggesting that the CD value is significantly advanced after two-month storage in the dark, associated with a higher monomeric anthocyanin concentration and a lower polymeric anthocyanin concentration [23]. It could be speculated that ultrasonic treatment involved the free anthocyanins composing and therefore caused the color density enhancement. 

Additionally, color tonality, expressed as the ratio of absorbance at 420 nm to that at 520 nm, was slightly increased by ultrasonic treatment at 40 kHz (Figure 1I). This increased T value after ultrasonic treatment was derived from the more blue-shifted components in wine, indicating the progress of polymerization between free anthocyanins and colorless polyphenols, which has also been reported in ultrasonic-treated red wine [25] and blueberry wine [10].

### 3.3. Ultrasonic Treatment Intensifies Anthocyanin Color Features

Given that wine color is chiefly determined by anthocyanins, in essence, deriving from the synergistic effect of copigmentation with free anthocyanins and polymeric pigments, the present research concentrated on the color values of anthocyanins to better understand the combined effects of ultrasonic and oak barrel treatment. The parameters WCP, FA, WC, CDR SO_2_, and CAW were all considered.

WCP denotes the total color of pigments after the conversion of colorless hemiketal anthocyanins and bisulfite adducts into the flavylium form, describing the color produced by both free and polymerized anthocyanins. Usually, the WCP value decreases with the aging time due to the progressive loss of monomeric anthocyanins [26]. As shown in Figure 2A, the present results indicate a higher WCP value of Sanhua plum wine after 28 kHz and 40 kHz ultrasonic treatment, indicating a reserved monomeric anthocyanin as the primary contributor. This is consistent with our previous study employing low-frequency ultrasonic treatment on blueberry wine, which also demonstrated a slightly increased WCP value [10]. It is worth noting that free anthocyanins defined by FA value were unvaried under different frequency processing levels in this study (Figure 2B), indicating the causal role of the polymerized anthocyanins in WCP performance. Homoplastically, the WC value referring to the absorbance at 520 nm after disintegrating anthocyanin bisulfite adducts, is another anthocyanin color indicator positively correlated to the copigmentation that arises from the interaction between anthocyanins and phenolic acids or flavanols. Based on the significantly amplified WC value, the accessible copigmentation enhancement appeared after ultrasonic treatment (Figure 2C). The continuously released partners from oak barrels, such as phenolic acids and flavanols, supposedly participated in chromogenic reactions, such as anthocyanin–tannin polymerization [27]. Meanwhile, in the bottle-aged wine, the WC value was maintained tightly [26], and no promotion was observed after ultrasonic treatment in the reported study [10]. Together, these data reveal that the wine color enhancement driven by ultrasonic treatment can be attributed to copigmentation.

During fermentation and aging, SO_2_ provides an antimicrobial property that is irreplaceable during winemaking [7]. Disadvantageously, free anthocyanin and some derivatives are reversibly hydrated and bleached by SO_2_. The concomitant colorless and unstable anthocyanin bisulfite product is associated with wine color loss, and is more critically engaged in hypochromic wine discoloration. However, certain anthocyanin derivatives with C-4 substituted and blocked structures hinder the hydration reaction, making it relatively stable to SO_2_ [7]. Therefore, CDR SO_2_ is considered the crucial chroma value to represent the group of stable anthocyanins and other derivatives or pigments during maturation. After three-month aging, ultrasonic at 28 kHz and 40 kHz conferred wine samples a stronger resistance to sulfur dioxide than the non-ultrasonic group (Figure 2D). This result indicates the increase in non-discolourable pigments in wine bodies that underwent ultrasonic and storage. To classify the non-discolourable pigments, we next calculated the value of CAW, as the proportion of WC assignable to CDR SO_2_. CAW is a classic and reliable index to assess the degree of aging. The precise storage conditions, such as container, temperature, humidity, as well as duration, will ultimately determine the CAW improvement. Therefore, CAW raises steadily with an increase in storage time, and this tendency could potentially indicate the formation of pyranoanthocyanin [28]. However, conducting ultrasonic treatment is not the decisive contributor to CAW promotion, as shown by the results presented in Figure 2E, which is in line with our previous study [10]. In fact, the acceleration of pyranoanthocyanins generation by ultrasonic treatment still lacks reliable evidence supporting the real wine system. Thus, it could be speculated that the color stability enhancement after ultrasonic treatment may not be driven by the pyranoanthocyanins, and that copigmentation prevails in determining the color of treated wine.

**Figure 3:**
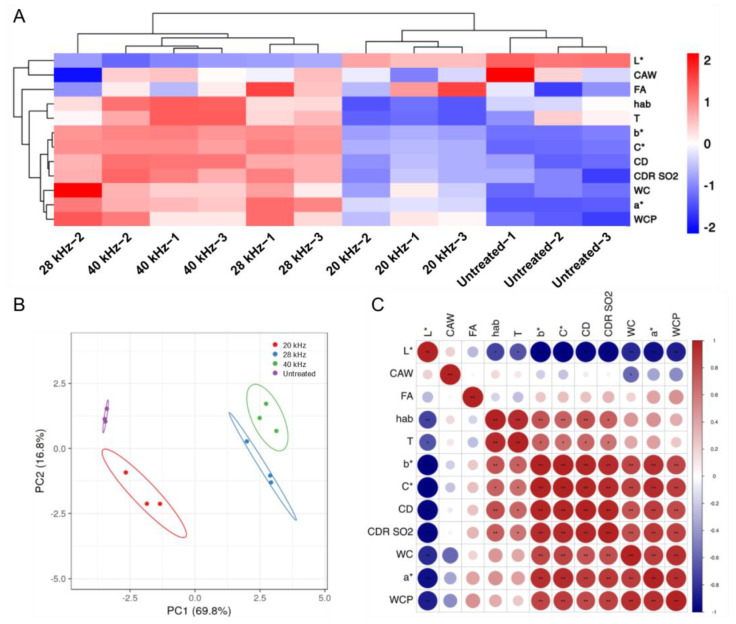
Clustering analysis and principal component analysis for the Sanhua plum wine and the correlation of different color parameters in Sanhua plum wine. (**A**) Clustering analysis for Sanhua plum wine with or without ultrasonic treatment after three-month aging considering the normalized indexes comprising the L*, a*, b*, *hab*, C*, WCP, WC, CD, T, and CDR SO_2_. (**B**) Principal component analysis. (**C**) Correlation of different color parameters. * *p* < 0.05, ** *p* < 0.01, indicating a significant correlation between the two parameters.

To comprehensively define the visible changes in color presentation and the hidden covariation between these color parameters in ultrasonic-treated Sanhua plum wine, we conducted a principal component analysis considering all the abovementioned color indexes. As shown in Figure 3A,B, wine samples after different treatments were well clustered and clearly distinguished from the untreated group, and the wine that experienced ultrasonic at 28 kHz and 40 kHz performed similar characteristics. This discrepancy proves the remarkable positive effect of ultrasonic treatment on Sanhua plum wine aging. In addition, the a* value was positively related to CD, CDR SO_2_, WC, and WCP, and the b* value was positively related to T, CD, CDR SO_2_, WC, and WCP (Figure 3C). Together, these results imply the objective correlation between color presentation and anthocyanins action in the wine body, and the changed anthocyanins profiles could be considered as one of the chief causes of sustaining satisfactory Sanhua plum wine color after ultrasonic treatment.

### 3.4. Ultrasonic Treatment Retards Anthocyanin Degradation

**Figure 4:**
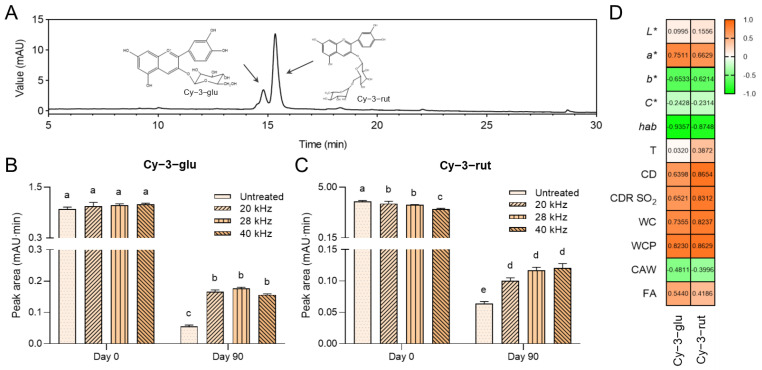
Anthocyanin changes during wine aging. (**A**) HPLC chromatogram of freshly fermented Sanhua plum wine at 520 nm. Two primary anthocyanins were identified as indicated. (**B**) Peak area of Cy-3-glu at 520 nm with different ultrasonic treatments. (**C**) Peak area of Cy-3-rut at 520 nm with different ultrasonic treatment. (**D**) The correlation between anthocyanins and color parameters. The number inside of the box indicates the coefficient of association. Different letters indicate a significant difference between the groups.

Anthocyanin as the primary pigment is responsible for Sanhua plum wine color, and is a highly reactive constituent on account of its unstable structure and diversified external factors, including enzymes, oxygen, metal ions, light, sulfur dioxide, and so on. Thus, anthocyanin degradation and sedimentation during fermentation and aging directly result in a decoloration effect. In the freshly fermented Sanhua plum wine, two major anthocyanins comprising cyanidin-3-*O*-glucoside (Cy-3-glu) and cyanidin-3-*O*-rutinoside (Cy-3-rut) were identified according to UV-Vis spectra (Figure 4A), mass spectra (Supplementary Figure S1), and the literature [3,29]. Moreover, no newly formed anthocyanin derivatives were found after three months of aging, and there were also no other differentiated substances except for Cy-3-glu and Cy-3-rut between untreated and treated wine under chromatography at 280 nm, 420 nm, and 620 nm. Thus, the reason for b*, CD, and WCP value upregulation could be explained by copigmentation.

After ultrasonic treatment, there is no instantaneous content change of Cy-3-glu in the wine sample. However, after three-month aging, the untreated wine expressed a significantly reduced Cy-3-glu level compared with ultrasonically treated wine (Figure 4B). Concerning Cy-3-rut, ultrasonic directly reduced Cy-3-rut in a frequency-dependent manner. However, paralleled with the reinforced Cy-3-glu, ultrasonic treatment maintained a higher level of Cy-3-rut when compared with the untreated wine (Figure 4C). The internal association between these two anthocyanins and color properties is summarized in Figure 4D. The remarkable correlation revealed the importance of both Cy-3-glu and Cy-3-rut in color performance.

Usually, ultrasonic-activated cavitation effects accompanied by free radicals and local high temperatures have been verified as severe threats anthocyanin stability [13,14]. The formed hydroxyl radicals or transitional H_2_O_2_ could engage in the cycloreversion reaction of anthocyanins, followed by the promotion of chalcone generation [30]. However, it is worth noting that the structural collapse of anthocyanins under ultrasonic stress is frequency-dependent, which means the low frequency has no negative impact on the anthocyanins [10]. In the present study, the employment of a plate-type ultrasonic producer reduced the local stress and helped the energy to scatter throughout the wine system, and the superimposed circulating fluid mode was also conducive to temperature reduction and avoiding locally continuous stimulation in a short time. Thus, only a slight reduction was found in Cy-3-rut, and most of the anthocyanins survived after ultrasonic treatment (Figure 4B,C).

During storage in model systems, Cy-3-rut is more stable than Cy-3-glu under different pH levels, temperatures, and cofactors due to the stabilizing effects conferred by additional glycosylation on inhibiting the formation of unstable intermediates by hydrolysis of the glycosidic component [31]. However, from our data, Cy-3-rut exhibits more frequency-dependent susceptibility to ultrasonic than Cy-3-glu, showing an immediate reduction in wine solution after experiencing ultrasonic waves (Figure 4B,C). Moreover, during wine aging, the complex systems and constituents cooperatively resulted in a more unstable decrease in Cy-3-rut than Cy-3-glu. These anomalies elucidate the extra factors that lead to anthocyanin degradation aside from the influence of pH and temperature. Especially, when including the oak barrel micro-oxygen environment, activated oxidative enzymes prevail in exacerbating anthocyanin degradation as the primary mechanism [32]. Ultrasonic treatment as a non-thermal processing technology induces the inactivation of polyphenol oxidase and peroxidase [33]. Discrepancies in the observed effects were classified by anthocyanin levels in the wine body, in which there was a greater reserve of anthocyanins in recipient wine submitted to ultrasonic treatment. This long-term desirable suppression of anthocyanin degradation could be partly deciphered by the irreversibly destroyed oxidase after an ultrasonic attack. However, the residue oxidase also performed a significant role in anthocyanins oxidation, thus the b* value gradually increased, associated with a yellow tonality that may also represent quinone generation, based on the fact that there was no pyranoanthocyanins formation. It should be noted that over-treatment of ultrasonic is detrimental to anthocyanin due to the direct destruction of the anthocyanin structure.

Overall, considering the invariable nature of the copigments, the enhanced copigmentation was largely derived from the surviving anthocyanins after ultrasonic treatment. Cy-3-glu and Cy-3-rut as the main contributors to color performance in Sanhua plum wine were protected under the beneficial effects mediated by ultrasonic treatment.

### 3.5. Ultrasonic Treatment Inactivates Polyphenol Oxidase

**Figure 5:**
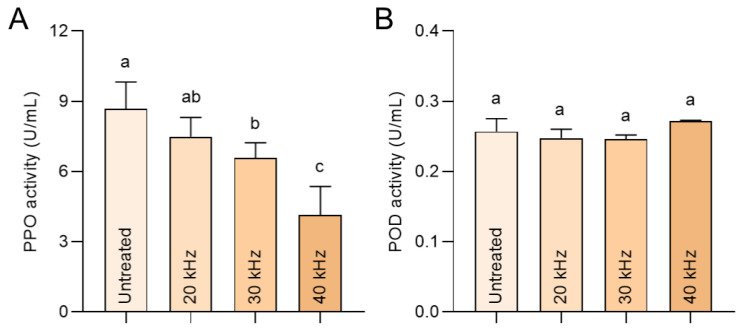
Polyphenol oxidase and peroxidase activity. (**A**) Polyphenol oxidase (PPO) and peroxidase (POD). (**B**) Activity of Sanhua plum wine after three-month aging. Different letters indicate a significant difference between the groups.

It has been well demonstrated that ultrasonic can chemically damage proteins, thus partially inhibiting polyphenol oxidase and peroxidase; this may directly contribute to monomeric anthocyanin degradation, and indirectly accelerate polymerization through the oxidation from polyphenols to quinone forms [34]. Moreover, the ultrasonic frequency is positively related to the inactivation of oxidase [33]. Clearly, there is a strong frequency-dependent effect on CD value alteration based on our data (Figure 1H), which could be interpreted by the passivation of detrimental enzymes targeted to anthocyanin disintegration. Considering the more reserved Cy-3-glu and Cy-3-rut inside the wine after ultrasonic treatment, we tried to decipher this objective result by mechanism combined with the activity of enzymes deleterious to anthocyanin stability. As shown in Figure 5A, endogenous polyphenol oxidase activity of wine, as the major driver of anthocyanin destruction [35], was dramatically decreased in an ultrasonic frequency-dependent manner. Meanwhile, another critical enzyme, peroxidase, was unaffected after different ultrasonic treatments (Figure 5B), which may be due to the insufficient ultrasonic conditions required for POD inactivation. Together, ultrasonic treatment can induce the frequency-dependent inactivation of PPO, which is responsible for anthocyanin protection.

### 3.6. Ultrasonic Treatment Strengthens the Antioxidative Capacity

Anthocyanins are important in determining the antioxidative capacity of fruit wine, which is responsible for multiple bioactivities in health improvement, such as cardiovascular disease, cancer, and neurodegeneration [36]. In fact, anthocyanins can interrupt oxidative chain reactions through scavenging free radicals, consequently diminishing oxidative stress [37]. Given the changed anthocyanin profile following ultrasonic treatment, an antioxidative capacity analysis was conducted on the final samples. Three different free radicals, DDPH·, ABTS^+^·, and O_2_·, were incorporated in this study to obtain a comprehensive profile of the antioxidative capacity of Sanhua plum wine.

**Figure 6:**
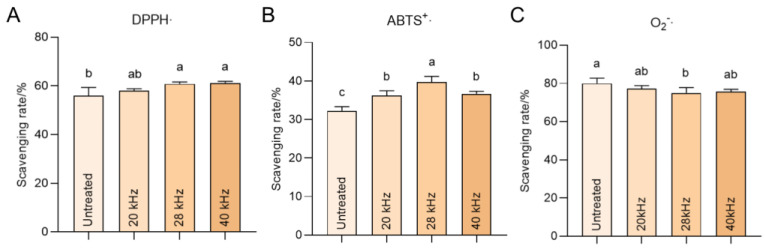
Antioxidative capacity of Sanhua plum wine under different ultrasonic treatment in vitro. The scavenging rates of DPPH· (**A**), ABTS^+^· (**B**), and O_2_^−^· (**C**) were in vitro tested in Sanhua plum wine after three-month aging. Different letters indicate a significant difference between the groups.

DPPH radical scavenging activity is most commonly applied to the antioxidative assessment of polyphenols, in which the transformation of the hydrogen atom from an antioxidant to DPPH could determine the antioxidant ability [38]. As shown in Figure 6A, ultrasonic treatment at 28 kHz and 40 kHz can slightly increase the DPPH scavenging rate of wine. ABTS radicals can be reduced in the presence of hydrogen-donating antioxidants [39]. In the present study, all trial frequencies positively contributed to an anti-ABTS^+^· ability (Figure 6B). However, considering the superoxide anion free radicals that completely differ from the cationic radicals, the frequency at 28 kHz exhibited slightly decreased anti-O_2_^−^· ability, and other conditions could not change quenching of this anion free radical (Figure 6C). These results collectively demonstrate that the increase in hydrogen-donating antioxidants plays a leading role in determining the antioxidative capacity of the ultrasonic-treated wine samples.

Generally, anthocyanins can donate one hydrogen atom from a hydroxyl group to free radicals, thus explaining their powerful antioxidative capacity. Meanwhile, antioxidative activity largely relies on the basic structural orientation of predominant anthocyanins, and not all of these are adept at diverse free radical cleaning [40]. For instance, it has been documented that delphinidin is more active against the superoxide anion than cyanidin, and pelargonidin is most efficient against hydroxyl radicals in all anthocyanin structures [40]. In a more complicated blueberry wine system composed of thirteen anthocyanin species, cyanidin content is relatively poor. Consequently, although the higher anthocyanin concentration was confirmed after ultrasonic treatment, there was no amelioration in the scavenging activity against DPPH· and ABTS^+^·, while a considerable promotion in clearing superoxide anion was proposed [10]. Thus, the antioxidative ability change under ultrasonic treatment is highly correlated with the composition of anthocyanins and other polyphenols.

Together, the selective antioxidation protection from proper ultrasonic treatment in Sanhua plum wine mainly involved in the nondegradative anthocyanins, according to their unique hydrogen donating ability based on their structural composition.

## 4. Conclusions

Induced by anthocyanin degradation, unavoidable decoloration is the major concern during winemaking and aging, particularly with regard to fruit wines with limited concentrations of inherent anthocyanins. The present study indicates the positive role of low-frequency ultrasonic treatment on Sanhua plum wine color retainment during aging in oak barrels. Using a plate-type ultrasonic producer, an ultrasonic frequency at 28 kHz and 40 kHz is prone to hold a stronger and more desired color performance, as indicated by our finding that the a*, b*, and C* values all significantly increased. Moreover, the color intensity dramatically improved in a frequency-dependent manner after three-month aging compared to the ultrasonic-free sample. By tracing the color composition, we found that the changed anthocyanin-related color parameters uncovered the crucial role of intensive copigmentation in determining the ultimate color. More importantly, ultrasonic treatment suppressed polyphenol oxidase activity associated with reduced anthocyanin degradation, and antioxidative capacity was accordingly protected in Sanhua plum wine after three-month aging. In conclusion, ultrasonic technology could be employed after fermentation with the intention of wine color improvement during oak barrel aging.

## Data Availability

Data is contained within the article or supplementary material.

## Supplementary Materials

The following supporting information can be downloaded at: https://www.mdpi.com/article/10.3390/foods11162435/s1, Figure S1: The secondary mass spectrum of anthocyanins fraction from HPLC. (A) Cyanidin-3-*O*-glucoside, Cy-3-glu. (B) Cyanidin-3-*O*-rutinoside, Cy-3-rut.
