# Supplementary material for: Color Stability Enhancement and Antioxidation Improvement of Sanhua Plum Wine under Circulating Ultrasound
Source: Foods. 2022 Aug 13;11(16):2435. doi: 10.3390/foods11162435 (PMC9407089; doi:10.3390/foods11162435)
Supplement: Supplementary file 1 [file foods-11-02435-s001.zip › foods-1817131-supplementary.pdf]

Supplementary

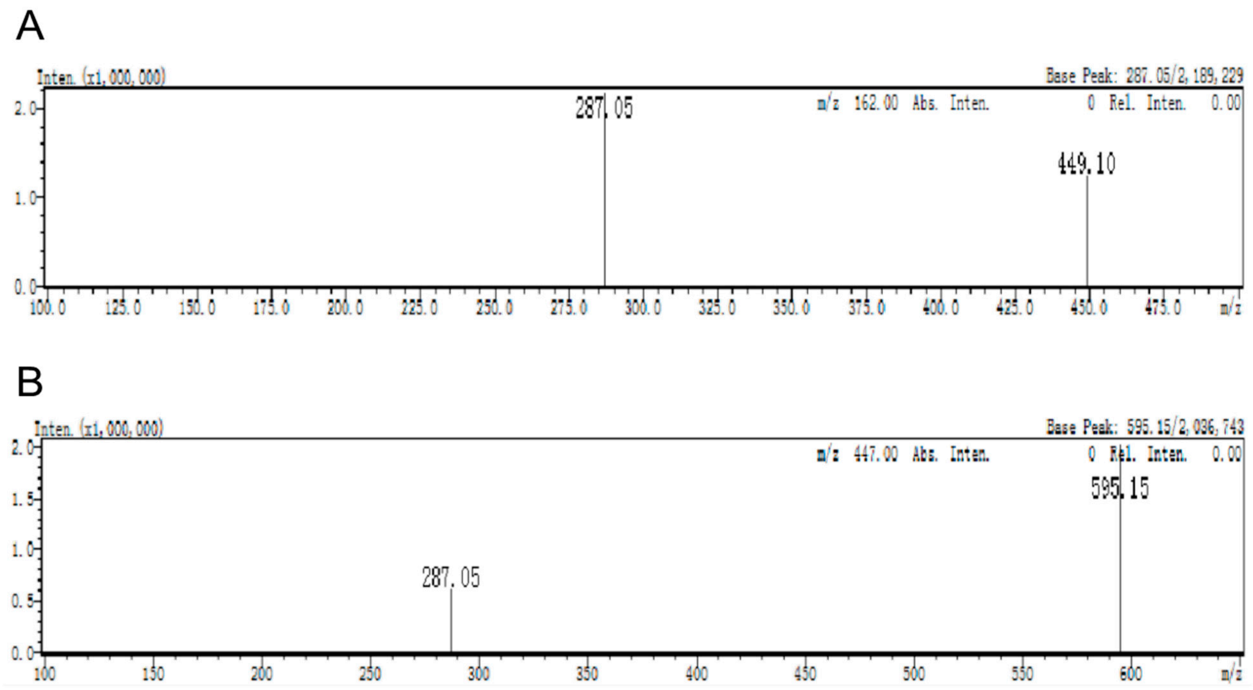

Figure S1. The secondary mass spectrum of anthocyanins fraction from HPLC. **(A)** Cyanidin-3-O-glucoside, Cy-3-glu. **(B)** Cyanidin-3-O-rutinoside, Cy-3-rut.
